# Amyloid-*β*-Induced Dysregulation of AMPA Receptor Trafficking

**DOI:** 10.1155/2016/3204519

**Published:** 2016-03-17

**Authors:** Sumasri Guntupalli, Jocelyn Widagdo, Victor Anggono

**Affiliations:** Clem Jones Centre for Ageing Dementia Research, Queensland Brain Institute, The University of Queensland, Brisbane, QLD 4072, Australia

## Abstract

Evidence from neuropathological, genetic, animal model, and biochemical studies has indicated that the accumulation of amyloid-beta (A*β*) is associated with, and probably induces, profound neuronal changes in brain regions critical for memory and cognition in the development of Alzheimer's disease (AD). There is considerable evidence that synapses are particularly vulnerable to AD, establishing synaptic dysfunction as one of the earliest events in pathogenesis, prior to neuronal loss. It is clear that excessive A*β* levels can disrupt excitatory synaptic transmission and plasticity, mainly due to dysregulation of the AMPA and NMDA glutamate receptors in the brain. Importantly, AMPA receptors are the principal glutamate receptors that mediate fast excitatory neurotransmission. This is essential for synaptic plasticity, a cellular correlate of learning and memory, which are the cognitive functions that are most disrupted in AD. Here we review recent advances in the field and provide insights into the molecular mechanisms that underlie A*β*-induced dysfunction of AMPA receptor trafficking. This review focuses primarily on NMDA receptor- and metabotropic glutamate receptor-mediated signaling. In particular, we highlight several mechanisms that underlie synaptic long-term depression as common signaling pathways that are hijacked by the neurotoxic effects of A*β*.

## 1. Introduction

Alzheimer's disease (AD) is the most common cause of dementia among the aging population. Early memory deficits and progressive loss of higher cognitive functions are common clinical features of AD patients. Pathologically, AD is characterized by insoluble aggregates of extracellular amyloid-beta (A*β*) peptides (senile plaques) and intracellular filaments composed of hyperphosphorylated tau (neurofibrillary tangles) in the brain. Strong evidence from human genetics and transgenic mouse models has implicated A*β* in the etiology and pathogenesis of AD [1]. A*β* peptides are derived from *β*-secretase- and *γ*-secretase-mediated sequential proteolytic cleavage of the amyloid-precursor protein (APP), with A*β*_1–40_ and A*β*_1–42_ being the most abundant species [2]. Many human mutations associated with familial AD, such as those that are found in genes encoding APP and the catalytic subunit of *γ*-secretase, presenilin (PS1 and PS2), promote amyloidogenic processing of APP, leading to enhanced A*β* production [3]. Recent studies have shown that soluble oligomeric forms of A*β* (ranging from dimers and trimers to dodecamers) exert potent and acute neurotoxic effects on the structure and function of synapses, including reduced excitatory synaptic transmission, loss of dendritic spines, and aberrant neuronal network activity [4, 5]. These deleterious effects could contribute to the cognitive deficit and memory loss associated with AD, indicating that “synaptic failure” is likely to be one of the earliest events that occurs in the pathogenesis of AD prior to neuronal loss [6–8].

The majority of fast excitatory synaptic transmission in the mammalian central nervous system is mediated by the release of glutamate from the presynaptic terminal and its binding to glutamate receptors on the postsynaptic membrane. The ionotropic glutamate receptors consist of AMPA (*α*-amino-3-hydroxy-5-methyl-4-isoxazolepropionic acid), NMDA (*N*-methyl-*D*-aspartate), and kainate receptors. Among these, AMPA receptors (AMPARs) are the principal receptors that mediate fast excitatory synaptic transmission in the mammalian brain. They are tetrameric assemblies of two dimers of four potential subunits (GluA1–GluA4) encoded by distinct genes,* GRIA1*–*GRIA4*. The predominant AMPARs expressed in the hippocampal and cortical pyramidal neurons are composed of GluA1/GluA2 and GluA2/GluA3 subunits [9]. Brief periods of high neuronal activity open NMDA receptors (NMDARs) and induce Ca^2+^ influx, leading to a long-lasting increase in synaptic efficacy, known as long-term potentiation (LTP), which is characterized by an increase in the number of AMPARs on the postsynaptic membrane and spine growth. In contrast, repetitive low frequency stimulation leads to the removal of synaptic AMPARs to produce long-term depression (LTD), that is, a decrease in synaptic strength. It has long been postulated that these forms of synaptic plasticity represent a cellular correlate of learning and memory [10].

One of the key mechanisms underlying synaptic plasticity is the tight control of AMPAR number at synapses. This requires a balance between the biosynthesis (number of receptors being produced), membrane trafficking (the movement of receptors to and from the plasma membrane via exocytosis and endocytosis), and degradation of receptors (receptor turnover), all of which are dynamically regulated by AMPAR interacting proteins as well as by various posttranslational modifications that occur on their cytoplasmic carboxyl terminal domains [11, 12]. Aberrant trafficking of AMPARs usually leads to impaired synaptic plasticity and deficits in learning and memory [11]. Importantly, several studies have demonstrated a role for A*β* in promoting AMPAR endocytosis and hence synaptic depression [13–16]. This review focuses primarily on NMDAR and metabotropic glutamate receptor- (mGluR-) mediated signaling. In particular, we highlight several mechanisms that underlie synaptic LTD as common signaling pathways that are hijacked by the neurotoxic effects of A*β*. Several pharmacological agents that target these pathways and are efficacious in inhibiting or reversing the neurotoxic effects of A*β* on glutamatergic neurotransmission and synaptic plasticity are also discussed.

## 2. A***β*** Alters Synaptic Plasticity* In Vitro* and* In Vivo*

The ability of neurons to modulate their synaptic strength is widely believed to be a cellular correlate of learning and memory. NMDAR-dependent LTP and LTD are two major forms of synaptic plasticity that are best studied in the hippocampus, a region of the brain that is both critical for memory formation and highly vulnerable to A*β* toxicity. It is well established that synthetic soluble A*β* oligomers [17, 18] or those secreted from cell lines overexpressing APP [19] acutely and potently block hippocampal LTP at high concentration. More recent studies have further shown that soluble A*β* dimers, but not A*β* monomers, either prepared by chemical cross-linking or extracted directly from postmortem AD brains, are extremely potent in inhibiting hippocampal LTP both* in vitro* and* in vivo* [4, 20]. Congruent with the LTP hypothesis of long-term memory, injection of these soluble A*β* oligomers into the rat hippocampus disrupts cognitive function and learned behavior [4, 21]. Most transgenic AD mouse models overexpressing different familial AD mutations, such as Tg2576 (APP_Swe_; K670N/M671L), PDAPP (APP_Ind_; V717F), 3xTg (APP_swe_, Tau P301L, and PS1 M146V), and 5xFAD (APP_swe_, APP_Florida_; I716V, APP_London_; V717I, PS1 M146L, and PS1 L286V), generally display impairments in LTP and cognition [22–26]. Notably, some AD transgenic mice show abnormal LTP and learning deficits well in advance of plaque formation [22, 27, 28]. Collectively, these results lend support to the idea that soluble oligomeric A*β* plays a key role in disrupting synaptic plasticity. More importantly, studies performed in human subjects have also revealed deficits in LTP-like cortical plasticity in mild-to-moderate AD patients [29–31].

Consistent with the fact that A*β* induces an impairment in LTP, soluble A*β* oligomers have been demonstrated to facilitate the expression of LTD in the hippocampus [4, 17, 32]. Although the exact mechanisms underlying A*β*-induced LTD remain equivocal, they have been shown to involve internalization of NMDA- and AMPA-type glutamate receptors, dendritic spine shrinkage, and eventual synaptic loss [14, 16, 33, 34].

## 3. Mechanisms Underlying A***β***-Induced Deficits in AMPAR Function

**Figure 1:**
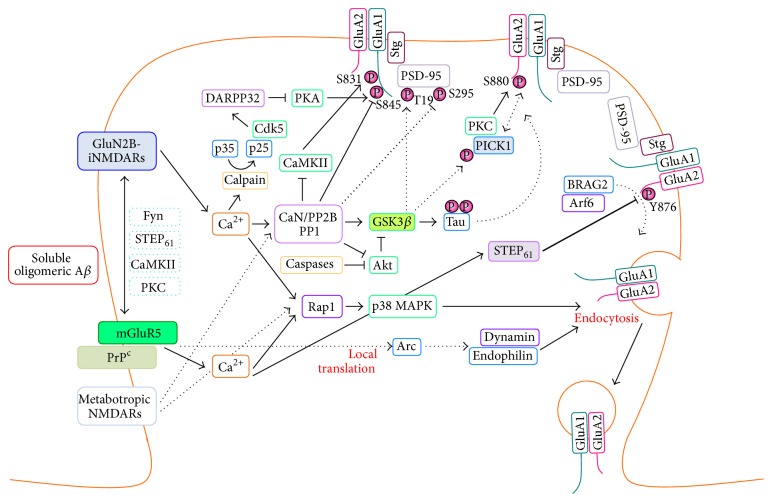
Signaling mechanisms involved in A*β*-induced AMPAR internalization. Soluble A*β* oligomers activate ionotropic NMDA (iNMDA) and metabotropic glutamate (mGlu) receptors, leading to an increase in intracellular Ca^2+^. Ca^2+^ subsequently activates a number of signal transduction cascades involving protein phosphatases (calcineurin, PP1, and STEP_61_) and protein kinases (Cdk5, PKC, and GSK3*β*) to modulate the phosphorylation of AMPAR subunits, as well as intracellular signaling and scaffolding molecules. Activation of these pathways, which are commonly shared with LTD, promotes AMPAR internalization and synaptic depression. The cross talk between NMDAR and mGluR5 signaling can be modulated by factors such as Fyn, CaMKII, PKC, and STEP_61_. The involvement of metabotropic NMDARs in mediating the neurotoxic effects of A*β*, which do not involve the flux of Ca^2+^, has recently been proposed, albeit this remains controversial. Dotted arrows indicate events that are inferred from the study of LTD and have not been shown to be directly involved in A*β*-mediated signaling. Thicker lines indicate common pathways, while colored boxes indicate potential therapeutics targets for AD.

Dynamic trafficking of AMPARs to and from synapses is a critical mechanism underlying the induction of synaptic plasticity. Defects in the endocytosis and lysosomal trafficking pathways are known to contribute significantly to AD pathogenesis [35]. Consistent with this notion, overexpression of APP and a high concentration of soluble oligomeric A*β* are able to induce the removal of surface AMPARs at synapses, leading to synaptic depression and inhibition of LTP [14, 19, 36, 37]. Mechanistically, these neurotoxic effects of A*β* are mediated by high levels of glutamate at synapses as a result of a disrupted glutamate reuptake process [32] that subsequently leads to aberrant activation of NMDARs, mGluRs, and the cellular prion protein (PrP^C^), as well as elevated levels of AMPAR ubiquitination. Activation of these signaling pathways in turn promotes synaptic depression, via common pathways shared with LTD as summarized in Figure 1, which are discussed in detail in the following sections.

### 3.1. NMDARs

NMDAR-dependent LTD induced by low frequency stimulation or by direct application of NMDA (chemically induced LTD) triggers Ca^2+^ entry into the postsynaptic compartment and activates protein phosphatase 2B (PP2B, also known as calcineurin), which in turn leads to the activation of protein phosphatase 1 (PP1) [38, 39]. PP1 and PP2B are known to mediate NMDAR-induced AMPAR internalization by dephosphorylating the GluA1 subunit of AMPARs at Ser-845 [40, 41], a protein kinase A (PKA) site that is crucial for maintaining the stability of AMPARs at perisynaptic sites and LTP [42–44]. NMDAR-dependent LTD also induces the p38 mitogen activated protein kinase (p38 MAPK) signaling pathway via the activation of Rap small GTPases, leading to the removal of AMPARs [45, 46].

Emerging evidence demonstrates that toxic levels of A*β* aberrantly enhance the activity of NMDARs in favor of LTD induction, thereby preventing LTP [32, 37, 47]. In cultured neurons and acute brain slices, soluble oligomeric A*β* induces excessive influx of Ca^2+^ through the GluN2B-containing extrasynaptic NMDARs, which subsequently activates the Rap-p38 MAPK signaling pathway, as well as the protein phosphatases, PP1 and calcineurin [13, 14, 16, 32, 37, 48–50]. One of the consequences of A*β*-induced activation of calcineurin is reduced phosphorylation of Ser-845, which induces AMPAR endocytosis and impairs the synaptic incorporation of these receptors [16]. Consistent with this finding, APP_Swe,Ind_ transgenic mice display lower levels of Ser-845 phosphorylation, a phenomenon that correlates well with the loss of AMPARs on the cell surface and deficits in initial learning and memory [16].

Another key substrate of PP1 the activity of which is required for the expression of NMDAR-dependent LTD is glycogen synthase kinase-3*β* (GSK3*β*) [51]. PP1 can activate GSK3*β* by a direct dephosphorylation mechanism, as well as via the modulation of the upstream caspase–Akt signaling pathways, which are also crucial for AMPAR internalization and LTD [51, 52]. Interestingly, both GSK3*β* and caspases are enzymes that have been widely implicated in AD. Indeed, it has been demonstrated that inhibition of LTP by A*β* is mediated by the caspase 3, Akt1, and GSK3*β* signaling pathway [32, 53]. Paradoxically, however, GSK3*β* activity has also been reported to play a role in maintaining AMPAR synaptic expression under basal conditions as its inhibition leads to the loss of surface AMPAR expression by controlling the rate of AMPAR internalization [54]. However, during NMDAR-dependent LTD, GSK3*β* may preferentially phosphorylate other substrates including the key scaffolding protein in excitatory synapses, postsynaptic density-95 (PSD-95). PSD-95 stabilizes AMPARs at synapses through its interaction with transmembrane AMPAR regulatory proteins (TARPs), auxiliary subunits of AMPARs [55]. Overexpression of PSD-95 promotes synaptic maturation and enhances synaptic strength, whereas PSD-95 knockdown results in the opposite effects [56–60]. It appears that GSK3*β* phosphorylation of PSD-95 at Thr-19, following its dephosphorylation at Ser-295 by PP1, destabilizes and mobilizes PSD-95 away from the PSD, resulting in increased AMPAR internalization [61, 62]. Whether or not the phosphorylation status of PSD-95 is modulated by oligomeric A*β* via the GSK3*β* and PP1 signaling pathways remains to be determined.

GSK3*β* is also a major kinase that phosphorylates the microtubule-associated protein tau [63, 64]. A*β* causes tau hyperphosphorylation and mislocalization from axons to somatodendritic compartments, where it accumulates and mediates A*β*-induced downregulation of surface AMPARs [65–68]. Recent studies have shown that NMDAR-induced GSK3*β* phosphorylation of tau at Ser-396 is required for hippocampal LTD by enhancing the interaction between the GluA2 subunits of AMPARs with the protein interacting with C-kinase 1 (PICK1) [69, 70], a process that is fundamental for AMPAR internalization and/or intracellular retention during LTD [71–76]. Furthermore, phosphorylation of PICK1 by GSK3*β* at Ser-416 has also been reported to augment this interaction [77].

GluA2 can be phosphorylated by protein kinase C (PKC) at Ser-880 and by the protein tyrosine kinase of the sarcoma (Src) family at Tyr-876, both of which are required for AMPAR internalization and LTD [78–80]. GluA2 phosphorylation at these sites differentially regulates the interaction of the subunit with PICK1 and glutamate receptor interacting proteins (GRIP) 1 and 2 [80, 81]. GRIP1 plays an important role in stabilizing AMPARs at synapses and is essential for LTD [72, 79]. Given that phosphorylation of GluA2 weakens the interaction of the subunit with GRIP1, but not PICK1, it has been postulated that LTD involves destabilization and detachment of GluA2 from synapses, allowing AMPARs to be internalized. In accord with the role of A*β* in inducing aberrant AMPAR endocytosis, one study has observed that oligomeric A*β* increases PKC-mediated phosphorylation of GluA2 at Ser-880 and subsequently reduces surface expression of AMPARs in cultured hippocampal neurons [15]. More importantly, several molecular and pharmacological manipulations that inhibit GluA2 internalization potently prevent A*β*-induced synaptic depression and rescue memory impairment in AD mice. These include the GluA2-R845A mutant [14], GluA2-3Y peptides [82], and a small molecule PICK1 inhibitor [83].

A new mechanism underlying the pathological action of A*β* that involves the cyclin-dependent kinase 5- (Cdk5-) activating peptide, p25, has recently been described by Seo et al. [26]. Elevated levels of p25 have been implicated in many neurodegenerative diseases, including AD [84]. In their study, Seo et al. found that A*β* induces calpain-mediated cleavage of p35 into p25 in the hippocampus, a process that requires the activity of GluN2B-containing NMDARs and Ca^2+^/calmodulin-dependent protein kinase II (CaMKII). The A*β*-induced elevation in p25/Cdk5 activity subsequently enhances the phosphorylation of dopamine- and cyclic adenosine monophosphate-regulated neuronal phosphoprotein (DARPP-32) at Thr-75, thereby inhibiting the activity of PKA [85]. In a synergistic manner, A*β* also triggers dephosphorylation of DARPP-32 at Thr-34, presumably by calcineurin, thereby releasing its inhibition on PP1 [86, 87]. These converging mechanisms eventually lead to the loss of GluA1 phosphorylation at Ser-845 and induce AMPAR internalization and synaptic depression. Remarkably, genetic inhibition of p25 generation rescues LTP and memory deficits in 5xFAD transgenic mice [26].

In addition to promoting the internalization of AMPARs, oligomeric A*β* can also act through mechanisms that prevent the forward trafficking of AMPARs towards the plasma membrane. A*β* has been shown to cause aberrant redistribution of CaMKII from the synaptic to the cytosolic fraction both in cultured neurons and in the brain of APP_swe_ transgenic mice [88]. CaMKII can potentiate AMPAR-mediated transmission via (a) phosphorylation of GluA1 at Ser-831 to enhance AMPAR channel conductance, (b) phosphorylation of the TARP, stargazin, to facilitate synaptic recruitment of AMPARs, and (c) potentiation of the Ras-ERK (extracellular signal-regulated kinase) pathway to promote AMPAR insertion into the plasma membrane [45, 89–91]. Consistent with the role of CaMKII in synaptic potentiation, exposure of soluble A*β* oligomers reduces surface GluA1 clusters in cultured neurons, concomitant with decreased AMPAR synaptic responses in cortical pyramidal neurons recorded from acute brain slices of APP_swe_ transgenic mice [88].

A*β* has been shown to interact with NMDARs [92, 93] and to reduce their surface expression through endocytosis [33]. A*β*-induced internalization of NMDARs involves dephosphorylation of the GluN2B subunit at Tyr-1472 by STEP_61_ (striatal-enriched protein tyrosine phosphatase 61), the expression of which is upregulated in several AD mouse models, as well as in the postmortem prefrontal cortex of AD patients [33, 94–96]. The fact that A*β* enhances the internalization of NMDARs seems counterintuitive given the role of NMDARs in mediating AMPAR endocytosis, spine loss, and ultimately excitotoxicity in neurons. Recent studies on the putative oligomeric A*β* receptor, PrP^C^, have provided insights into two potential mechanisms that regulate NMDAR function [97, 98]. Firstly, soluble oligomeric A*β* binding to PrP^C^ activates the tyrosine kinase Fyn, which initially phosphorylates GluN2B and transiently enhances NMDAR function, before the STEP_61_ level increases and dephosphorylates GluN2B [99]. Secondly, A*β* disrupts the ability of PrP^C^ to limit excessive NMDAR activity in a copper-dependent manner, potentially by chelating copper ions and preventing them from binding to PrP^C^, thereby producing large nondesensitizing steady-state NMDAR currents [100]. Albeit controversial, loss of PrP^C^ function has been reported to prevent A*β*-induced LTP and memory impairment in mice [98, 101–105]. Despite this, the role of PrP^C^ in regulating AMPAR trafficking has not been directly examined.

Recent studies by Kessels and colleagues have challenged the central role of NMDAR-mediated Ca^2+^ influx in A*β*-induced synaptic depression [106]. It is well established that the neurotoxic effects of oligomeric A*β* on synapses can be blocked by the NMDAR antagonist, D-APV (D-2-amino-5-phosphonopentanoic acid), which prevents glutamate binding and blocks the activation of NMDARs. However, noncompetitive NMDAR antagonists that block ion flow through the receptor, such as MK-801, ketamine, and 7-chlorokynurenic acid, are not able to rescue A*β*-mediated synaptic depression [106, 107]. A similar finding was recently reported for oligomeric A*β*-induced dendritic spine loss [108]. Consistent with the idea that A*β* operates through shared pathways with LTD, metabotropic, but not ionotropic, NMDAR function has been shown to be required for NMDAR-dependent LTD in the hippocampus by activating the p38 MAPK signaling pathway [109]. In fact, ligand binding to the extracellular domain of NMDARs induces conformational change and movement of their cytoplasmic tails, allowing PP1 to dephosphorylate CaMKII together with other signaling molecules that contribute to synaptic depression [110, 111]. Although the role of metabotropic NMDARs remains controversial [46], it does offer an explanation for the fact that the FDA-approved NMDAR antagonist, memantine, has poor efficacy in treating early-stage AD [112]. Further research is warranted, as delineating the metabotropic NMDAR signaling pathway may shed light on new strategies for the development of future AD drugs.

### 3.2. mGluRs

mGluRs belong to the G-protein-coupled receptor superfamily that modulates neuronal excitability, synaptic transmission, and plasticity in the central nervous system [113]. Group I mGluRs, which consist of two members, mGluR1 and mGluR5, predominantly localize to the postsynaptic membrane and are canonically coupled to G*α*_q/11_ to activate phospholipase C*β* (PLC*β*) that catalyzes the hydrolysis of phosphoinositides into inositol 1,4,5-triphosphate (IP_3_) and diacylglycerol (DAG). Subsequently, these second messengers trigger the release of Ca^2+^ from intracellular stores and activate PKC, respectively. Group I mGluRs, and more specifically mGluR5, are the predominant receptors that mediate mGluR-dependent LTD in the hippocampus and have been widely implicated in AD [114].

It is well established that mGluR-dependent LTD requires the internalization of GluA2-containing AMPARs, leading to a long-term reduction in the number of surface AMPARs [115, 116]. One of the mechanisms that regulates mGluR-induced AMPAR endocytosis involves the phosphorylation of GluA2 at Ser-880 by PKC, a process that is facilitated by PICK1 [117–119]. However, in the CA1 region of the hippocampus, internalization of AMPARs does not require PKC but instead relies on the dephosphorylation of GluA2 at Tyr-876 by STEP_61_ [120–122]. Dephosphorylation of GluA2 stimulates the binding of BRAG2 (brefeldin resistant Arf GEF 2), which in turn activates the small GTPase Arf6 through augmentation of its GEF (guanine-nucleotide exchange factor) activity and promotes AMPAR endocytosis [122]. In accordance with this model, it has been reported that A*β*-induced internalization of AMPARs requires STEP_61_ activity [95]. Genetic deletion of STEP_61_ restores the number of AMPARs on the postsynaptic membrane, enhances LTP, and improves cognitive function in AD mice [95, 123]. A new small molecule inhibitor of STEP_61_, TC-2153, has recently been shown to reverse cognitive deficits in 3xTg AD mice [124]. Like NMDAR-dependent LTD, mGluR-mediated LTD also involves the Rap1-p38 MAPK signal transduction pathway to facilitate AMPAR internalization via the formation of the GDI-Rab5 complex [125–127]. In addition, a role for ERK in mGluR-dependent LTD has also been reported [128].

One unique feature of mGluR-dependent LTD is its requirement for rapid translation of preexisting mRNAs (local protein translation) in dendrites [129]. mGluR-dependent* de novo* protein synthesis can be regulated through multiple pathways, including the PI3K-Akt-mTOR (mammalian target of rapamycin) and ERK signaling pathways that converge on the initiation and elongation factors of protein translation [130]. Several mRNA encoding proteins that regulate AMPAR trafficking are locally translated during mGluR-dependent LTD, including the activity-regulated cytoskeleton-associated protein (Arc), microtubule-associated protein 1B (MAP1B), and STEP [121, 131–133]. All of these proteins are known to facilitate the internalization of AMPARs. MAP1B is a known GRIP1 binding protein [134]. Given that GRIP1 stabilizes AMPARs at synapses, the newly synthesized MAP1B may sequester GRIP, hence loosening its interaction with GluA2. On the other hand, Arc interacts with the endocytic proteins, endophilin and dynamin, and is able to enhance dynamin polymerization and GTPase activity, thereby promoting AMPAR endocytosis [135, 136]. Interestingly, soluble oligomeric A*β* rapidly induces Arc expression in neurons, which may contribute to the loss of AMPARs from the plasma membrane [137]. Moreover, Arc also regulates the endosomal trafficking of APP and BACE1, as well as PS1, a mechanism that is essential for the activity-dependent production of A*β* in the brain, and genetic deletion of Arc reduces the A*β* load in APP_swe_;PS1ΔE9 transgenic AD mice [138]. This may serve as a positive feedback mechanism underlying the overproduction of A*β* in the pathophysiology of AD.

Studies from several laboratories have implicated the mGluR-dependent signaling pathway in the neurotoxic effects of A*β* on synaptic function [4, 14, 139–143]. Notably, genetic and pharmacological inhibition of mGluR5 prevents oligomeric A*β*-induced impairment in LTP, spine loss, and cognitive deficits in AD mouse models [139, 142–145]. More recently, a seminal study by Strittmatter and colleagues identified an interaction between mGluR5 and PrP^C^, which together act as a coreceptor for oligomeric A*β* [144]. They also revealed an essential role for mGluR5 and PrP^C^ coupling in the pathology of AD [146]. Mechanistically, mGluR5 links PrP^C^ to key intracellular signaling molecules, such as Homer1b/c, Pyk2, Fyn, and CaMKII, all of which play major roles in synaptic plasticity [144, 146, 147]. When neurons are exposed to oligomeric A*β*, the PrP^C^-mGluR5 complex mediates the aberrant activation of Pyk2, Fyn, and CaMKII, causing altered neuronal states that lead to impaired LTP [99, 144, 146]. It is interesting to note that A*β* also induces a biphasic alteration in CaMKII activity, resembling that of Fyn, in a PrP^C^-mGluR5-dependent manner, and that this is accompanied by the increased association of mGlu5 with CaMKII [146]. Given that mGluR5 activation enhances NMDAR forward trafficking through CaMKII-mediated phosphorylation of GluN2B at Ser-1303 [148], it is hypothesized that A*β*-induced enhancement of the association between mGluR5 and CaMKII may prevent synaptic potentiation. Furthermore, pharmacological activation of mGluR5 in the presence of PrP^C^ causes a redistribution of CaMKII into the cytoplasm [147], which may have an impact on AMPAR trafficking.

Interestingly, the cross talk between mGluR5 and NMDAR signaling is bidirectional. Not only can mGluR5 potentiate NMDAR currents through CaMKII and PKC signaling pathways [148, 149], but also activation of NMDARs can potentiate mGluR5 responses under physiological conditions [150, 151]. This involves the NMDAR-dependent activation of calcineurin that dephosphorylates mGluR5 and reduces receptor desensitization. However, a high concentration of NMDA can induce PKC-dependent mGluR5 phosphorylation and inhibit mGluR5 responses [152]. Although the interaction of mGluR5 and NMDARs has been implicated in synaptic plasticity and various animal behaviors [153–156], their alteration in the presence of A*β* binding to PrP^C^ and how this impacts on AMPAR trafficking remain unclear.

### 3.3. Protein Ubiquitination

Posttranslational ubiquitination, a regulatory signal that controls protein trafficking and turnover, has recently emerged as an important mechanism that regulates AMPAR function [157, 158]. All AMPAR subunits undergo activity-dependent ubiquitination in cultured neurons, a process that is Ca^2+^-dependent and requires the activity of L-type voltage-gated Ca^2+^ channels [159–161]. The primary E3 ligases that catalyze the ubiquitination of GluA1 and GluA2 subunits are Nedd4-1 and RNF167, respectively [160, 162]. While the role of protein ubiquitination on the GluA1 and GluA2 subunits in ligand-induced AMPAR endocytosis remains controversial, it is well accepted that ubiquitination of AMPARs regulates the intracellular sorting of receptors into late endosomes for degradation [159–161, 163]. Under normal conditions, the degradation of AMPARs is required for protein homeostasis to ensure turning over of old or used receptors in order to maintain healthy levels of AMPARs in neurons. However, when the ubiquitin pathway is hijacked (e.g., by elevated levels of A*β*), there is an excessive downregulation of AMPARs and synaptic depression. Indeed, a new finding has demonstrated a role for naturally secreted and synthetic A*β* in promoting the ubiquitination of AMPARs by Nedd4-1 [164]. Interestingly, knocking down Nedd4-1 rescued A*β*-induced synaptic deficits, including reduced glutamatergic synaptic transmission, decreased levels of surface AMPARs, and the loss of dendritic spines. These findings have important implications in targeting ubiquitin E3 ligases as potential drug targets for the treatment of AD.

## 4. Concluding Remarks

Research over the past decade has provided strong evidence that the cognitive deficit associated with AD is caused by the neurotoxic effects of soluble A*β* oligomers on synaptic function. Increasing evidence indicates that the trafficking of AMPARs, which is essential to multiple forms of synaptic and structural plasticity in the brain, is aberrantly dysregulated by oligomeric A*β* and manifests as impairments in LTP, learning, and memory. It is particularly encouraging to learn that pharmacological and genetic manipulations that block endocytosis or enhance the forward trafficking of AMPARs can rescue LTP and reverse cognitive deficits in AD mice. Given that A*β*-induced AMPAR internalization requires the same adaptor proteins as the conventional trafficking pathway, it will be challenging to minimize unwanted side effects. Hence, further research is needed to identify specific targets for improving the memory deficits associated with AD. Rapid progress has been made in delineating the molecular mechanisms and signaling pathways underlying the loss of AMPARs from the plasma membrane induced by oligomeric A*β* (Figure 1). The discovery of PrP^C^ as a receptor for soluble A*β* oligomers that signals through NMDA and mGluR5 receptor has underscored the importance of glutamatergic signaling in the etiology of AD. It is likely that these receptors act cooperatively to mediate the synaptotoxic effects of A*β*, highlighting the need for further investigation of the associated signaling mechanisms with a view to developing more effective therapeutic strategies for the treatment of AD.
